# Virioplankton Assemblage Structure in the Lower River and Ocean Continuum of the Amazon

**DOI:** 10.1128/mSphere.00366-17

**Published:** 2017-10-04

**Authors:** Bruno S. de O. Silva, Felipe H. Coutinho, Gustavo B. Gregoracci, Luciana Leomil, Louisi S. de Oliveira, Adriana Fróes, Diogo Tschoeke, Ana Carolina Soares, Anderson S. Cabral, Nicholas D. Ward, Jeffrey E. Richey, Alex V. Krusche, Patricia L. Yager, Carlos Eduardo de Rezende, Cristiane C. Thompson, Fabiano L. Thompson

**Affiliations:** a Laboratory of Microbiology, Institute of Biology, Federal University of Rio de Janeiro (UFRJ), Rio de Janeiro, RJ, Brazil; b Laboratory of Advanced Production Management Systems-SAGE, COPPE-Technology Management Center-CT2, Federal University of Rio de Janeiro (UFRJ), Rio de Janeiro, RJ, Brazil; c Centre for Molecular and Biomolecular Informatics (CMBI), Radboud University Medical Centre, Radboud Institute for Molecular Life Sciences, Nijmegen, The Netherlands; d Department of Marine Sciences, UNIFESP Baixada Santista, Santos, SP, Brazil; e Center for Ecology and Socio-Environmental Development (NUPEM/UFRJ), Federal University of Rio de Janeiro (UFRJ), Macaé, RJ, Brazil; f Laboratory of Hydrobiology, Institute of Biology, Federal University of Rio de Janeiro (UFRJ), Rio de Janeiro, RJ, Brazil; g Marine Sciences Laboratory, Pacific Northwest National Laboratory, Sequim, Washington, USA; h School of Oceanography, University of Washington, Seattle, Washington, USA; i Center of Nuclear Energy in Agriculture (CENA), Federal University of São Paulo (USP), Piracicaba, SP, Brazil; j Department of Marine Sciences, University of Georgia, Athens, Georgia, USA; k Laboratory of Environmental Sciences, Darcy Ribeiro State University of Northern of Rio de Janeiro (UENF), Campos, RJ, Brazil; University of Michigan—Ann Arbor

**Keywords:** Amazon River, microbial communities, next-generation sequencing, river ecology, river plume, virome

## Abstract

The Amazon River watershed and its associated plume comprise a vast continental and oceanic area. The microbial activities along this continuum contribute substantially to global carbon and nutrient cycling, and yet there is a dearth of information on the diversity, abundance, and possible roles of viruses in this globally important river. The aim of this study was to elucidate the diversity and structure of virus assemblages of the Amazon River-ocean continuum. Environmental viral DNA sequences were obtained for 12 locations along the river’s lower reach (*n* = 5) and plume (*n* = 7). Sequence assembly yielded 29,358 scaffolds, encoding 82,546 viral proteins, with 15 new complete viral genomes. Despite the spatial connectivity mediated by the river, virome analyses and physical-chemical water parameters clearly distinguished river and plume ecosystems. Bacteriophages were ubiquitous in the continuum and were more abundant in the transition region. Eukaryotic viruses occurred mostly in the river, while the plume had more viruses of autotrophic organisms (*Prochlorococcus*, *Synechococcus*) and heterotrophic bacteria (*Pelagibacter*). The viral families *Microviridae* and *Myoviridae* were the most abundant and occurred throughout the continuum. The major functions of the genes in the continuum involved viral structures and life cycles, and viruses from plume locations and Tapajós River showed the highest levels of functional diversity. The distribution patterns of the viral assemblages were defined not only by the occurrence of possible hosts but also by water physical and chemical parameters, especially salinity. The findings presented here help to improve understanding of the possible roles of viruses in the organic matter cycle along the river-ocean continuum.

**IMPORTANCE** The Amazon River forms a vast plume in the Atlantic Ocean that can extend for more than 1,000 km. Microbial communities promote a globally relevant carbon sink system in the plume. Despite the importance of viruses for the global carbon cycle, the diversity and the possible roles of viruses in the Amazon are poorly understood. The present work assesses, for the first time, the abundance and diversity of viruses simultaneously in the river and ocean in order to elucidate their possible roles. DNA sequence assembly yielded 29,358 scaffolds, encoding 82,546 viral proteins, with 15 new complete viral genomes from the 12 river and ocean locations. Viral diversity was clearly distinguished by river and ocean. Bacteriophages were the most abundant and occurred throughout the continuum. Viruses that infect eukaryotes were more abundant in the river, whereas phages appeared to have strong control over the host prokaryotic populations in the plume.

## Abstract (precis)

The Amazon River forms a vast plume in the Atlantic Ocean that can extend for more than 1,000 km. Microbial communities promote a globally relevant carbon sink system in the plume. Despite the importance of viruses for the global carbon cycle, the diversity and the possible roles of viruses in the Amazonia are poorly understood. The present work assesses, for the first time, the abundance and diversity of viruses simultaneously in the river and ocean in order to elucidate their possible roles. DNA sequence assembly yielded 29,358 scaffolds, encoding 82,546 viral proteins, with 15 new complete viral genomes from the 12 river and ocean locations. Viral diversity was clearly distinguished by river and ocean. Bacteriophages were the most abundant and occurred throughout the continuum. Viruses that infect eukaryotes were more abundant in the river, whereas phages appeared to have strong control over the host prokaryotic populations in the plume.

## INTRODUCTION

The Amazon biome is the largest in Brazil, comprising a territory of 4,196,943 km^2^ (1). Its main river, the Amazon, is one of the longest in the world (6,992.06 km) (2), and it has the largest discharge (6.3 trillion m^3^/year) (3), with an average flow of 175 million liters of water per s (4), which corresponds to 20% of the world’s freshwater discharge in the ocean (5).

Upon reaching the ocean, the river forms a plume (a relatively shallow layer of hyposaline water) with a depth that varies from 5 m (6) to 25 m (7). The plume extends far off the shelf for more than 1,000 km (6, 8) during periods of high river water levels (May to June) (5, 9–11). The surrounding Amazon rainforest and wetlands enrich the river with organic matter (OM), some of which is either remineralized or carried to the ocean, forming a river-to-ocean continuum (5, 12, 13). Nutrients from the river foster a microbial community that enhances carbon sequestration in the ocean, forming a globally relevant carbon sink system that takes up ~28 Tg C/year (14). The plume also influences the recently discovered Amazon reef system, a mesophotic habitat dominated by coralline algae and sponges, rich in chemoautotrophic and anaerobic microbes (7). The ocean also influences the river, especially during periods of low water levels (November to December) (5, 9–11), when the force of ocean tides reverses the river flow near and upstream of the mouth (5, 15) and also reduces its level of discharge, an effect that can be detected in the river near the city of Óbidos (Pará state [PA]), which is located 900 km upstream of the river mouth (16). This region between the Óbidos and the river mouth is known as the lower Amazon River. The waters in the river channels near the cities of Belém (PA), Macapá (north and south; Amapá state), and Óbidos (PA) are brownish and turbid, with high concentrations of organic matter and suspended sediments (SS), while Tapajós River (PA), an Amazon tributary, has light green and more transparent waters, with lower levels of sediment (5, 17).

Viruses are abundant biological entities that perform key biological roles such as the following. (i) Regulation of microbial population numbers occurs by killing of the blooms of rapidly growing hosts through lytic virus infection, which leads to equilibrium in the levels of diversity of these hosts, as accounted for by the Kill-the-Winner theory (18). Recent discoveries have expanded this theory, including the discovery that phages prioritize lysogenic infections in ecosystems with high microbial densities, originating the Piggyback-the-Winner theory (19, 20). (ii) Promotion of genetic diversity occurs by viral transduction, which influences the functions of the hosts and of the ecosystem (21, 22). (iii) Increases in host metabolism occur by activation of viral auxiliary metabolic genes (AMGs) after infection to reprogram the host to produce more cellular resources to enable the dissemination of viral progeny (23, 24). (iv) Influences over carbon and organic matter cycles occur through the activity of the viral shunt (25), releasing particulate organic matter (POM) and dissolved organic matter (DOM) from lysed cells and thus providing nutrients for prokaryotes instead of leaving the host cell intact for grazing by eukaryotes (26–28). When DOM enters the microbial loop (29), levels of cellular respiration and of resulting CO_2_ outgassing may increase. Additionally, viral particles can be adsorbed to POM (30) and then sink, directly contributing to the deposition of organic matter into the sediment of aquatic systems, or can be advected, contributing to the export of organic matter from river to ocean.

The lack of sequences to which the raw reads can be assigned in reference databases often leads to poor virome annotation performance (31, 32). The building of specific databases through virome cross-assembly can circumvent this issue (20, 33) and also can improve understanding of the influence of environmental parameters on viral communities (20, 33, 34). The diversity and structure of viral assemblages in river systems worldwide, and particularly in the Amazon River, are poorly understood (35). Previous studies have addressed specific virus taxonomic groups by the use of PCR (36) and cultivation (37); all of those studies were restricted to a very limited geographical range in the Amazon region. Despite advances in our knowledge regarding the microbial diversity in the Amazon plume (7, 14, 38), only a few studies have addressed the role of microbes along the continuum, especially in the lower Amazon River (5, 12, 39), and the roles of viruses have been mostly overlooked. Thus, a more comprehensive understanding of the complete virus diversity along the Amazon continuum is lacking, especially in the river’s lower reaches and plume and also in the ecological context of environmental parameters (40).

The aim of this study was to elucidate the diversity and assemblage of planktonic viruses in the Amazon River-plume continuum. We performed the first broad viromics analysis of this system using a shotgun approach to define the major taxonomic and functional groups along the continuum and to characterize how environmental parameters, possible viral hosts, and geographical locations shape the composition of the viral assemblage in this vast and relevant geographic area.

## RESULTS

### Water physical-chemical and biological analyses.

The water physical-chemical parameters (Table 1) and biological parameters (cell and viral particle counts and chlorophyll values) (Table 2) that were investigated revealed distinct environmental conditions along the continuum. Principal-component analysis (PCA) of the physical-chemical data (see Fig. S1A in the supplemental material) revealed three major groups of samples: river samples, plume samples, and samples from a transition region between them, which is formed by locations near the river mouth (station 10 [St10] and St11). The lower river locations (Tapajós, Óbidos, north Macapá, south Macapá, Belém) were warmer, with higher concentrations of inorganic nutrients and dissolved organic carbon (DOC) and with lower pH and lower surface dissolved inorganic carbon (SurfDIC) and oxygen concentrations. The plume locations (St6, St4, St3, St1, and St15) exhibited strong temperature and salinity (Sal) gradients between the mouth and the outer region, with lower nutrient and organic matter concentrations overall and higher pH and higher SurfDIC and oxygen concentrations. Finally, the transition locations (St10 and St11) displayed intermediate values between the two extremes (Table 1 and Fig. S1A).

10.1128/mSphere.00366-17.2 FIG S1  (A and B) Principal-component analysis of the physical-chemical parameters of the water and the dinucleotide frequency of the complete viromes. (A) The covariance PCA was built with physical-chemical parameters of the water and showed river locations (brown), including the Óbidos (Obi), north Macapá (NMac), south Macapá (SMac), Bélem (Bel), and Tapajós (Tap); transitions (black), including St10 and St11; and plume (blue) groups, including St3, St4, St15, St1, and St6. Labels display the amounts of variance explained by each axis. (B) The covariance PCA was built with the dinucleotide frequency of the complete viromes and showed river locations (brown) plus transitions (black), including Bélem (Bel), Óbidos (Obi), north Macapá (NMac), south Macapá (SMac), and Tapajós (Tap) and the St10 and St11 transitions, and plume (blue) groups, including St4, St3, St15, St1, and St6. Download FIG S1, TIF file, 0.4 MB. Copyright © 2017 Silva et al. 2017 Silva et al. This content is distributed under the terms of the Creative Commons Attribution 4.0 International license.

**TABLE 1:** Water physical-chemical parameters of the river and plume of the Amazon River^a^

Parameter	Value											
	Tapajós	Óbidos	North Macapá	South Macapá	Belém	St10	St11	St6	St4	St3	St1	St15
Salinity	0.01	0.02	0.02	0.02	0.02	0.11	12.17	30.29	23.6	24.44	31.56	36.34
Water temp (°C)	30.1	29.2	29.5	29.3	29.6	28.7	28.5	28.04	29.0	29.27	28.44	28.4
pH	6.75	6.58	6.72	6.71	6.99	7.26	7.67	7.83	8.11	8.15	8.03	8.05
Partial pressure of carbon dioxide (PCO_2_) (µatm)	1,039	5,488	4,786	4,673	2,013	831	515	569	251	234	368	390
Surface dissolved inorganic carbon (µmol·C/kg)	113	464	502	484	339	415	818	1,643	1,372	1,409	1,774	2,030
Dissolved oxygen (mg O_2_/kg)	5.54	3.50	4.75	5.08	6.20	6.45	5.54	5.02	9.07	7.37	7.12	7.28
Saturation of dissolved oxygen (%)	95.3	44.7	62.3	66.6	81.7	83.3	76.0	78.0	136	112	111	118
Ammonium (µM)	1.47	0.72	1.35	1.35	3.12	0.18	0.014	0.00	0.003	0.23	0.00	0.00
Nitrate + nitrite (µM)	5.64	13.8	17.9	17.9	7.75	9.58	8.52	7.74	1.69	0.00	0.00	0.5
Phosphate (µM)	0.51	0.45	0.26	0.26	0.18	0.88	1.10	1.08	0.30	0.12	0.07	0.11
Silica (µM)	130	118	144	144	109	39.2	28.3	47.2	20.5	22.5	12.9	0.25
Dissolved organic carbon (µM)	346	381	308	308	244	277	191	72	127	93	87	61
Dissolved organic nitrogen (µM)	10.4	8.5	0.0	0.0	2.6	11.0	13.6	8.8	11.3	7.7	—	8.3
Dissolved organic phosphorus (µM)	—	—	—	—	—	0.4	0	0	0.3	0.3	—	0.3
Fine suspended sediment (FSS) (mg·liter^−1^)^b^	5.2	32.3	77.0	49.7	14.4	—	—	—	—	—	—	—
Particulate lignin (µg lignin liter^−1^)^b^	3.6	25.7	10.1	10.9	10.9	—	—	—	—	—	—	—

a Rivers, Tapajós, Óbidos, north Macapá, south Macapá, and Belém; plumes, St10, St11, St6, St4, St3, St1, and St15. —, no data available (no samples collected).

b Values are from Ward et al. (5).

**TABLE 2:** Microbial counts and chla concentrations of the water from river and plume of the Amazon River^a^

Parameter	Value											
	Tapajós	Óbidos	North Macapá	South Macapá	Belém	St10	St11	St6	St4	St3	St1	St15
Chlorophyll (µg·liter^−1^)	5.12	1.03	0.87	0.70	2.83	0.64	2.49	0.550	5.100	0.535	0.160	0.910
Virus (no. of particles × 10^6^ ml^−1^)	5.34	4.57	2.76	3.34	4.11	ND	ND	0.827	16.900	17.800	6.240	10.300
Bacteria (no. of cells × 10^6^ ml^−1^)	3.27	3.77	3.63	3.77	3.64	3.85	3.07	1.690	1.500	1.310	0.681	0.634
Virus-to-microbe ratio (VMR)	1.63	1.21	0.76	0.89	1.13	ND	ND	0.490	11.270	13.590	9.160	16.250
Picoeuk (cells × 10^3^ ml^−1^)	4.13	1.29	0.736	1.09	1.34	2.89	1.57	0.707	0.376	0.591	1.110	1.070
Nanoeuk (cells × 10^2^ ml^−1^)	25.4	2.99	1.22	3.62	9.03	0.783	1.26	7.590	2.710	1.590	0.000	0.285
*Prochlorococcus* (no. of cells × 10^4^ ml^−1^)	ND	ND	ND	ND	ND	ND	0.140	0.202	0.000	1.260	15.400	4.570
*Synechococcus* (no. of cells × 10^4^ ml^−1^)	18.1	0.465	0.281	0.326	1.23	ND	0.743	2.250	4.780	0.421	4.490	5.700

a Rivers, Tapajós, Óbidos, north Macapá, south Macapá, and Belém; plumes, St10, St11, St6, St4, St3, St1, and St15. chla, chlorophyll a; Picoeuk, autotrophic picoeukaryotes; Nanoeuk, autotrophic nanoeukaryotes; ND, not defined.

Viral particle abundance was higher in the plume, but bacterial abundance was higher in the river. Thus, virus-to-microbe ratios (VMR) were higher in the plume (with the exception of St6) than in the river (Table 2). The levels of chlorophyll, cyanobacteria, picoeukaryotes, and nanoeukaryotes corresponded to the different river origins, exhibiting higher values in the samples from the rivers from Brazil’s central region (Tapajós and Belém) and lower values in the samples from the main Amazon River course (Óbidos, north Macapá, and south Macapá); however, large variations were observed across the plume. The brownish waters of the main river course also had more fine suspended sediment (FSS) and particulate lignin than Tapajós and Belém (Table 2). During the sampling period, the Óbidos River showed a water level that was normal with respect to historical measured levels (5, 41) (Fig. S2).

10.1128/mSphere.00366-17.3 FIG S2  Amazon River discharge. Data represent Agência Nacional de Águas records of discharge from the Óbidos River; the sum of discharges from the Tapajós, Xingu, and Tocantins (Belém) rivers; and the sum of discharges from the Óbidos River plus the lowland rivers, which represents the total discharge of water to the Amazon River plume. The yellow box indicates the time period of sampling in the river and plume (12 July to 12 August), which corresponds to the period of falling water levels of the Amazon River. (Data derived from Ward et al. [5]). Download FIG S2, TIF file, 0.7 MB. Copyright © 2017 Silva et al. 2017 Silva et al. This content is distributed under the terms of the Creative Commons Attribution 4.0 International license.

### Virome yield and dinucleotide frequency analysis.

Virome sequencing yielded 146,022 (St11) to 2,964,975 (St4) reads, with a mean read size of 230 bp (±50 bp) and mean GC content level of 44% (±8.3%) (see Table S1 in the supplemental material). The PCA of dinucleotide frequency revealed two groups separated by PC1 (Fig. S1B). One was dominated by the river samples and consisted of Belém, north Macapá, south Macapá, Óbidos, and Tapajós and also of transition locations St10 and St11; the other was dominated by the plume samples and included St1, St3, St4, St6, and St15. The GC content of the river-dominated group was higher (49.4% ± 6.2%) than that of the plume-dominated group (36.4% ± 2.5%) (*P* = 0.0009 [*t* test]).

10.1128/mSphere.00366-17.8 TABLE S1  Sequence data after preprocessing and MG-RAST annotation, with classification at the domain level (percent), according to locations of the river and plume of the Amazon River. Rivers: Tapajós, Óbidos, north Macapá, south Macapá, and Belém. Plumes: St10, St11, St6, St4, St3, St1, and St15. SSU, ribosomal small subunit (SILVA database; https://www.arb-silva.de/); LSU, ribosomal large subunit (SILVA database); QC, quality control (MG-RAST). Download TABLE S1, DOCX file, 0.02 MB. Copyright © 2017 Silva et al. 2017 Silva et al. This content is distributed under the terms of the Creative Commons Attribution 4.0 International license.

### New viral genomes and proteins discovered in the Amazon viral community.

Virome cross-assembly resulted in 29,358 scaffolds longer than 1 kbp, amounting to 71.8 Gbp of data (N50 = 2,709). Among these, 15 were circular and longer than 10 kbp, likely representing new complete viral genomes. Together, the scaffolds encoded 82,546 proteins, but only 35,381 (43%) exhibited similarity to entries in the NCBI nr database (viruses, 13,158; bacteria, 21,103; archaea, 357; eukaryotes, 702; unclassified, 61), often with low identity levels (mean identity of 60% ± 22.5%), highlighting the novelty of this data set (Table S2). The proteins included both typical viral structural and information processing proteins (e.g., capsid proteins and DNA polymerases) and transduction and auxiliary metabolic proteins encoded by genes carried by viruses that are involved in diverse pathways that are important for host physiology (e.g., photosynthesis and nutrient transporters) (Table S2). The classification of Amazon scaffolds with VirSorter provisionally confirmed 3,266 to be viral sequences (for complete phage contigs, highly certain confirmation (pretty sure), 623; moderately certain confirmation (quite sure), 2,634; for prophages, highly certain confirmation, 1; moderately certain confirmation, 8), representing 11.12% of the total number of scaffolds obtained. In addition to 3,692 genomes from the literature, the custom database contained 6,958 sequences in total. The VirFinder analysis of the Amazon viral scaffolds validated by VirSorter returned a mean score of 0.79 ± 0.23 (mean *P* = 0.05 ± 0.09), while the VirFinder analysis of all Amazon scaffolds returned a mean score of 0.62 ± 0.31 (mean *P* = 0.15 ± 0.21).

10.1128/mSphere.00366-17.9 TABLE S2  Taxonomical and functional annotation of protein sequences encoded by Amazon viral assembled scaffolds. Download TABLE S2, XLSX file, 2.9 MB. Copyright © 2017 Silva et al. 2017 Silva et al. This content is distributed under the terms of the Creative Commons Attribution 4.0 International license.

The functional profile obtained with HUMAnN2 showed that genes related to biosynthesis of nucleosides and nucleotides were abundant in all locations (Fig. S3). The transition and the plume had a more diverse functional profile, including genes related to fatty acid and lipid biosynthesis and to respiration and other groups of genes not related to common viral functions. The river’s north Macapá and Belém locations did not return any results (Fig. S3).

10.1128/mSphere.00366-17.4 FIG S3  Functional profile of the Amazon viral scaffolds. Data represent a functional profile of relative levels of abundance, based on the annotation of genes with HUMAnN2. The gene functions were categorized according to the MetaCyc superclasses. The locations north Macapá (NMac) and Belém (Bel) returned no results. Rivers, Tapajós (Tap), Óbidos (Obi), and south Macapá (SMac); transitions, St10 and St11; plumes, St6, St4, St3, St1, and St15. Download FIG S3, TIF file, 0.3 MB. Copyright © 2017 Silva et al. 2017 Silva et al. This content is distributed under the terms of the Creative Commons Attribution 4.0 International license.

### Viral community abundance profiles.

**FIG 1:**
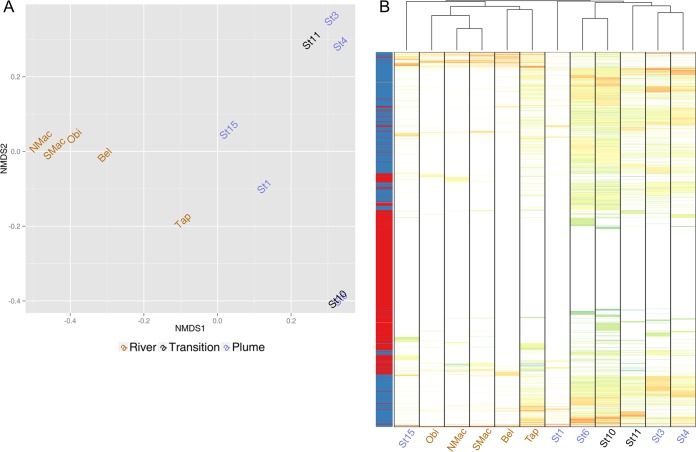
Nonmetric multidimensional scaling (NMDS) (A) and dendrogram and heat map (B) of the relative abundances of the data in the custom database across the Amazon viromes. The custom database is formed by the assembled Amazon scaffolds classified as viruses by VirSorter (blue rows in the leftmost column) plus the reference viral genomes (red rows). Cell coloring reflects relative abundances (log_10_ transformed for clarity). Both scaffolds and genomes were clustered based on the Manhattan distances between their distributions. (A) NMDS of the custom database showing the separation of the river group from the transition-plus-plume group. (B) Abundance profile of reads mapped to the custom database. The dendrogram denotes a blurred separation between river and plume, with St15 (plume) grouping in the river group and the transition (St10 and St11) grouping in the plume group. The colors of the labels represent the following elements: brown, rivers; black, transitions; blue, plumes.

The nonmetric multidimensional scaling (NMDS) of the abundance profile of the custom database in the Amazon viromes showed a separation of the rivers from the plume stations, according to NMDS axis 1 (Fig. 1A), indicating a separation of freshwater and saline waters, with the exception of St10 (a river mouth station), whose samples grouped with the saline samples. This pattern of separation by salinity was also observed in the PCA of the dinucleotide frequency profiles (Fig. S1B), with the exception of the samples from the brackish-water station (St11), which grouped with the riverine samples. The salinity influence was not detected in the dendrogram analysis of the same custom database, as the St15 plume grouped with the rivers and the other plume stations and the transition region formed another group (Fig. 1B). In addition, the heat map showed that the contribution of reference viral genomes in the Amazon viromes was lower than that seen with the Amazon scaffolds (Fig. 1B).

### Succession of possible viral hosts along the continuum.

**FIG 2:**
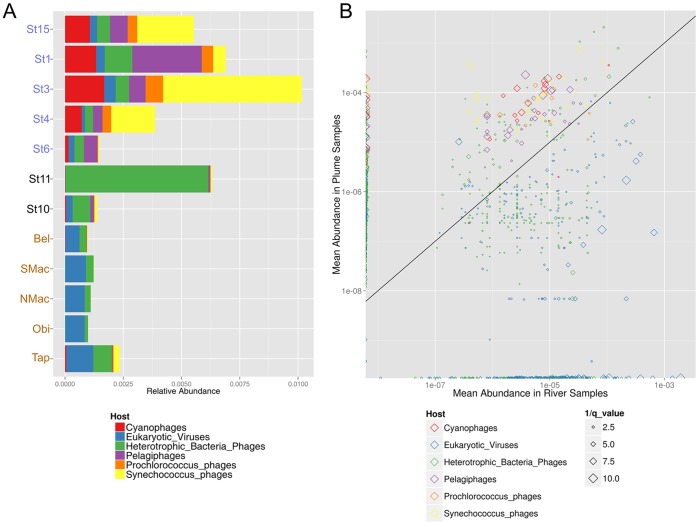
(A and B) Relative (A) and mean (B) abundances of reference viral genomes across the continuum summarized by host type. (A) The bar graph shows the relative abundances of the reference viral genomes according to their respective hosts. A succession of patterns from river to ocean is observed, where the river locations (brown), including Belém (Bel), north Macapá (NMac), south Macapá (SMac), Óbidos (Obi), and Tapajós (Tap), are dominated by viruses of eukaryotic organisms; the transitions (black), including transitions St10 and St11, show an increase in the levels of heterotrophic bacterial viruses; and the plumes (blue), including plumes St1, St3, St4, St6, and St15, possess more viruses that infect autotrophic organisms. (B) Scatterplot displaying the median abundances of sequences in samples from Amazon River (*x* axis) and plume (*y* axis). Each point represents a reference viral genome (color coded as described for panel A). The sizes of the points are inversely proportional to the false-discovery-rate (*q*) values, meaning that larger points display more-significant changes in abundance between the two sets of samples. Data corresponding to both axes are shown in log_10_ scale; the black line represents a 1:1 ratio.

The reference levels of viral genome abundance along the continuum showed distinctive patterns according to their hosts (Fig. 2A): in riverine samples (Tapajós, Óbidos, north Macapá, south Macapá, and Belém), viruses of eukaryotes (e.g., pandoraviruses, megaviruses, and mimiviruses) were more abundant; in the transition plume (St10 and St11), phages of heterotrophic bacteria increased in abundance accompanied by a decrease in the abundance of eukaryotic viruses; and in the plume (St6, St4, St3, St1, and St15), a trend of more cyanophages, prochlorophages, and synechophages than phages of heterotrophic bacteria was observed, with the exception of St6 and St1, where pelagiphages had higher relative abundance. Analysis of the individual abundance patterns of the reference viral genomes corroborated this pattern (Fig. 2B). The majority of cyanophages, pelagiphages, prochlorophages, and synechophages were more abundant in the plume than in the river, whereas eukaryotic viruses and most phages infecting heterotrophic bacteria (other than *Pelagibacter* sp.) were more abundant in the river. In these analyses, pelagiphages, prochlorophages, and synechophages were separated from the common groups because of the abundance and importance of their respective hosts in marine waters: "*Candidatus* Pelagibacter ubique" (42), *Prochlorococcus*, and *Synechococcus* (43).

### Viruses most important for river and plume separation.

**FIG 3:**
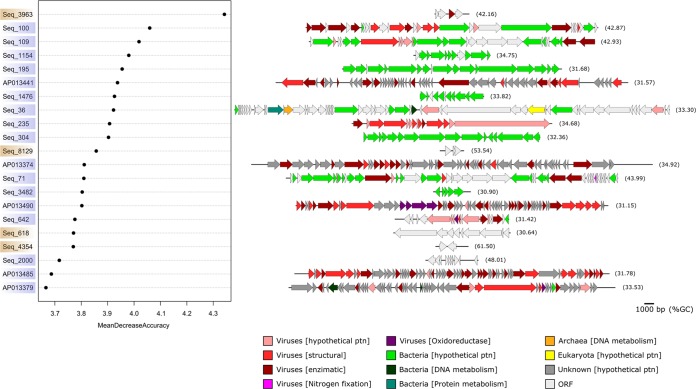
The Amazon viral scaffolds and viral genomes most important for river and plume segregation. Data represent the 16 Amazon viral scaffolds (Seq_3963 et al.) and 5 viral genomes from the literature (AP013441 et al.) that were most abundant (relative abundance) and important for river (riverine; light brown) and plume (oceanic; light blue) segregation, according to a random forest analysis. Identifiable genes in these sequences are represented and categorized according to the taxonomic domain and its general function. The %GC content is also indicated for each sequence. ORF, open reading frame with no similarity to any entry in the GenBank nr protein database; ptn, protein.

The random forest analysis identified 21 viral sequences (corresponding to 16 VirSorter-validated Amazon scaffolds and 5 reference genomes) whose abundance was most important for river and plume separation (Fig. 3). Four Amazon scaffolds were more abundant in the river (riverine), while the 17 others abounded in the plume (oceanic) (Fig. 3). Amazon scaffold Seq_3963 (riverine) was the scaffold most indicative of river-plume separation. This scaffold corresponds to a replication-associated protein from a sewage-associated circular DNA virus, representing a protein family that is associated with single-stranded DNA (ssDNA) viruses of animals (*Circoviridae*) and plants (*Nanoviridae*, *Geminiviridae*) (Table S2). None of the other genes from the riverine scaffolds had similarity to genes encoding proteins listed in the GenBank nr protein database. Overall, the majority (77.7%) of the genes had no identifiable function, and the identifiable genes (22.3%) encoded proteins for cellular metabolism (DNA, proteins), especially from *Bacteria*. In addition, possible viral AMGs encoding proteins related to nitrogen fixation (one in Seq_71) and oxidoreductases [six in total, including two Fe(II)-dependent oxygenases and two tryptophan halogenases in AP013490 (uncultured Mediterranean phage), one thioredoxin in Seq_642, and one thioredoxin in AP013379 (uncultured Mediterranean phage)] were detected (Fig. 3 and Table S2).

### Viral richness and diversity in the continuum.

The Shannon diversity index data indicated higher diversity for some Amazon River samples (Óbidos, north Macapá, and south Macapá) and lower diversity for the remaining rivers, as well as for the plume locations (Table S3). This trend was corroborated by rarefaction curves inferred from the abundance profiles of these samples (Fig. S4), which revealed that Óbidos, north Macapá, and south Macapá were much further from reaching saturation than the remaining river and plume samples. The Simpson index data showed that the plumes corresponding to St6, St15, and St4 were the most dominant locations; the richness values indicated that plumes St4 and St3 and also transition St10 were richer (Table S3). The Shannon index of viral functions, annotated by the Metagenomic RAST server (MG-RAST), indicated lower values for the turbid rivers plus St10 (which also has turbid waters) (mean, 1.4 ± 0.2) and higher values for the plume plus St11 and the Tapajós River (clear water river) (mean, 2.8 ± 0.3) (Table S3).

10.1128/mSphere.00366-17.5 FIG S4  Rarefaction curves. Rarefaction curves were built based on the abundance profile of reads mapped on the complete set of assembled Amazon scaffolds plus the reference viral genomes. Download FIG S4, TIF file, 0.5 MB. Copyright © 2017 Silva et al. 2017 Silva et al. This content is distributed under the terms of the Creative Commons Attribution 4.0 International license.

10.1128/mSphere.00366-17.10 TABLE S3  Diversity indexes and richness. The data in the first three columns were calculated based on the abundance profile of mapped reads in reference phage genomes and assembled Amazon scaffolds. The last column lists the Shannon diversity index of viral functions based on the MG-RAST annotation. Rivers: Tapajós, Óbidos, north Macapá, south Macapá, and Belém. Plumes: St10, St11, St6, St4, St3, St1, and St15. Download TABLE S3, DOCX file, 0.01 MB. Copyright © 2017 Silva et al. 2017 Silva et al. This content is distributed under the terms of the Creative Commons Attribution 4.0 International license.

### Automated metagenome annotation: summary, classification, and canonical analysis of principal coordinates (CAP).

The number of validated sequences remaining after MG-RAST quality control (QC) was performed ranged from 133,414 (St11) to 1,519,118 (St4) (Table S1). These sequences were classified as corresponding to rRNA genes (with levels ranging from 0.48% in St11 to 7% in St4), annotated proteins (6.23% in St1 to 72.31% in St11), unknown proteins (26.6% in St11 to 86.28% in St1), and unknown sequences (0% to 4.52% in north Macapá) (Table S1). The number of sequences classified as small ribosomal subunits (SSU) ranged from zero in south Macapá to 206 (0.034% of the valid sequences) in St10, whereas the number of large ribosomal subunit (LSU) sequences ranged from zero in north Macapá, south Macapá, and Belém to 754 (0.124%) in St10 (Table S1). Of the total number of available annotated proteins, 0.3% (St11) to 61.9% (north Macapá) were classified as viruses at the domain level after MG-RAST annotation (Table S1).

The taxonomical classification of viral sequences at the family level indicated that a large fraction (11% to 20.6%) represented unclassified sequences of the viral domain (Fig. S5A). The most abundant identifiable families were *Microviridae* (9.58% to 18.18%) and *Myoviridae* (6.08% to 17.18%). Other abundant families were *Circoviridae*, *Podoviridae*, *Phycodnaviridae*, and *Siphoviridae* (Fig. S5A). The functional classification of subsystems at level 1 (collections of functionally related protein families) (44) showed that sequences of phage, prophage, transposable element, and plasmid (PPTP) subsystems (which included viral gene sequences of capsid, neck, tail, packaging machinery, phage replication, and phage lysins, among others) were most abundant in all locations but were even more abundant in the turbid rivers and St11 (transition) (mean abundance of 72.4% ± 20.8%) than in St10 (transition), plume, and Tapajós (16.9% ± 5.5%) (Fig. S5B). Other less-abundant subsystems (such as cofactors, vitamins, prosthetic groups, and pigments; regulation and cell signaling; cell wall and capsule; photosynthesis; and others) were detected only in the transition, plume, and Tapajós.

10.1128/mSphere.00366-17.6 FIG S5  (A and B) Taxonomic and functional profiles based on the automated annotation of the Amazon viromes. (A and B) Graph bars indicating the relative abundances of the viral families/groups (A) and the functional subsystems (level 1) (B), according to the MG-RAST annotation for each location. Legend: River locations (brown), Tapajós (Tap), Óbidos (Obi), north Macapá (NMac), south Macapá (SMac), and Belém (Bel); transitions (black), St10 and St11; plumes (blue), St6, St4, St3, St1, and St15. Download FIG S5, TIF file, 1.1 MB. Copyright © 2017 Silva et al. 2017 Silva et al. This content is distributed under the terms of the Creative Commons Attribution 4.0 International license.

Canonical analysis of principal coordinates (CAP) revealed a river-dominated group and a plume-dominated group (Fig. S6). The river-dominated group was formed by north Macapá, Óbidos, south Macapá, Belém, and St11 (transition); it exhibited higher values for nitrate plus nitrite (NO_3_ + NO_2_), partial pressure of carbon dioxide (PCO_2_), dissolved organic carbon (DOC), silica (SiO_2_), and water temperature (WTemp) and included more *Circoviridae*, *Geminiviridae*, and *Nanoviridae* (all ssDNA viruses). The plume-dominated group included St4, St3, St15, St1, St6, Tapajós (clear river), and St10 (transition), with a greater contribution of saturation of dissolved oxygen (SatDO), pH, salinity (Sal), surface dissolved inorganic carbon (SurfDIC), and dissolved organic nitrogen (DON) and an enrichment of *Poxviridae*, *Mimiviridae*, *Microviridae*, *Siphoviridae*, *Phycodnaviridae*, *Myoviridae*, *Iridoviridae*, and *Podoviridae*, as well as unclassified sequences of *Caudovirales* and virus. A permutational multivariate analysis of variance (PERMANOVA) test indicated that Sal (*P* < 0.001) was the most important parameter for the river and plume separation, followed by pH, PCO_2_, and SurfDIC (*P* < 0.01) (Fig. S6).

10.1128/mSphere.00366-17.7 FIG S6  Relationship between annotated viral taxonomic profile and physical-chemical parameters. Canonical analysis of principal coordinates (CAP) of the viral families and abundance (according to the MG-RAST annotation) against a constraints matrix of physical-chemical data, from the 12 studied locations of the Amazon, was performed. A river-dominated (north Macapá [NMac], Óbidos [Obi], south Macapá [SMac], Belém [Bel], St11) group and a plume-dominated (St10, St4, St3, St15, Tapajós [Tap], St6, St1) group were evidenced, with the major parameters Sal, pH, PCO_2_, and SurfDIC structuring the virioplankton assemblages. A PERMANOVA test was used to calculate the statistical significance of the CAP ordination data. Colors of the locations: rivers, brown; transitions, black; plumes, blue. Viral families/groups (red): *Geminiviridae* (Gem), *Nanoviridae* (Nan), *Circoviridae* (Cir), *Alloherpesviridae* (Allo), *Marseilleviridae* (Mar), *Herpesviridae* (Her), *Poxviridae* (Pox), unclassified derived from virus (unVir), *Microviridae* (Mic), unclassified derived from caudovirales (unCau), *Podoviridae* (Pod), *Iridoviridae* (Iri), *Mimiviridae* (Mim), *Siphoviridae* (Sip), *Phycodnaviridae* (Phy), *Myoviridae* (Myo). Water parameters (gray): nitrate plus nitrite (NO_3_ + NO_2_), partial pressure of carbon dioxide (PCO_2_), dissolved organic carbon (DOC), silica (SiO_2_), water temperature (Wtemp), saturation of dissolved oxygen (SatDO), surface dissolved inorganic carbon (SurfDIC), pH, salinity (Sal), and dissolved organic nitrogen (DON). Download FIG S6, TIF file, 0.3 MB. Copyright © 2017 Silva et al. 2017 Silva et al. This content is distributed under the terms of the Creative Commons Attribution 4.0 International license.

## DISCUSSION

### The structures of the virioplankton assemblages are distinct between the river and plume of the Amazon.

Despite the continuum formed by the Amazon River extending from land to ocean, the river and plume represent different ecosystems, characterized by distinct patterns of viral assemblages and water parameters and separated by a transition plume formed by locations St10 and St11. This trend was clearly observed with the PCA of the physical-chemical parameters, for which the river ecosystem was characterized by higher levels of respiration and organic matter (DOC). In contrast, the plume ecosystem demonstrated more photosynthetic processes and the production/release of organic nitrogen forms (DON), indicating waters that are more autotrophic and oligotrophic, a pattern that corroborates the results of a previous study (14). This pattern is reinforced by the increased presence of genes of photosynthesis in the plume, as observed in the functional profiles of HUMAnN2 and MG-RAST. The transition displayed features that were intermediate between those of the river and plume (e.g., low salinity and SurfDIC, like the rivers, and lower temperature and PCO_2_, like the plume).

The virioplankton data corroborate the main separation of the river and plume ecosystems, as observed with the %GC content and the dinucleotide frequency of the viromes as well as with the annotation-dependent approaches: the mapped profile of viral abundance (NMDS and dendrogram analysis), the abundance and distribution of possible hosts inferred from the reference viral genomes, the most abundant and important viral scaffolds and genomes in the river and plume, and the CAP ordination of the viral families. A few exceptions were observed, as in the case of the grouping of St15 with the rivers in the dendrogram, probably caused by the lack of some Amazon viral scaffolds discarded by VirSorter. This program can be less sensitive in analyzing small viral genomes with few predicted genes (45), an important limitation considering the great abundance of small ssDNA viruses observed in the Amazon continuum. However, the good VirFinder scores obtained from all Amazon scaffolds and from the VirSorter-validated ones indicate that our viral database is reliable. The grouping of Tapajós (clear water river) with the plumes in the CAP might represent selection of groups from similar hosts (e.g., cyanophages, prochlorophages, and synechophages) and their related viruses, as these locations possess similar environmental conditions, such as higher light penetration, which favors photosynthetic organisms. The occurrence of genes related to photosynthesis subsystems in Tapajós and in the plume reinforces this hypothesis. Considering the Óbidos River water level to be a proxy that is representative of the whole continuum and since conditions were normal during the sampling period, it is expected that the pattern presented here can be reproduced during the periods of falling water levels in the Amazon River.

Environmental parameters may regulate the virioplankton community structure when these viral particles are free in the water (46). Factors such as temperature, salinity, pH, UV light, and nutrients (nitrogen, phosphorous) can interact directly, enhancing or reducing virion viability in marine environments (22). In the Amazon continuum, the most important parameters for structuring virioplankton assemblages were Sal, pH, PCO_2_, and SurfDIC, according to the viral CAP results. Although PCO_2_ has been found to be related to viral and bacterial abundances in an Amazon tributary (40), the possible direct effects of the presence of gaseous and dissolved forms of CO_2_ in virions remain unknown. The influence of geographical location and environmental conditions, such as salinity, on marine virioplankton has been well documented (46–48). The patterns presented here suggest that intrinsic physical-chemical and biological parameters of the water bodies along the Amazon continuum may have a major impact on the viral community composition, leading to patterns of separation of viral groups and possible hosts, thus shaping the similarities among geographical locations.

A clear shift in viral assemblage composition occurred along the river-plume continuum, where the viruses were grouped according to the reference phage host types. Riverine samples (Tapajós, Óbidos, north Macapá, south Macapá, and Belém) were dominated by eukaryotic viruses, likely as a consequence of the elevated concentrations of autotrophic nanoeukaryotes and picoeukaryotes measured at those sites as well as of the larger heterotrophic protists and of the land contribution of plant and animal cells. At the transition zone, phages that infect heterotrophic bacteria became increasingly abundant, while the abundance of eukaryotic viruses declined. The widespread changes in environmental conditions in this zone may lead to the selection of more-tolerant organisms such as heterotrophic bacteria and their viruses. Toward the ocean, the abundances of bacteria and microalgae decreased, but the abundances of cyanobacterial and viral particles drastically increased, leading to enrichment of the waters in phages of cyanobacteria and also of *Pelagibacter*. This pattern of possible viral hosts is reinforced by the eukaryotic sequences, where rivers contained 9% of the reads and 60% of the transcripts (39), in contrast to an overall lower contribution of eukaryotic reads in the plume (38).

### The majority of the members of cosmopolitan viral families are bacteriophages.

The widespread occurrence of *Microviridae* (small ssDNA phages) indicates that their hosts may have a similarly broad distribution, surviving throughout the continuum, as reinforced by the occurrence of phages of heterotrophic bacteria (*Microviridae*, *Myoviridae*, *Podoviridae*) along the continuum. Although some genetically similar viruses are widespread, most viruses are constrained to specific environmental conditions where their hosts can survive and reproduce (26). The high abundance of bacteriophages in Amazon freshwaters is consistent with a previous report (49).

Tailed viruses have been reported to be more resistant to changes in ionic strength (22, 50). In addition, bacteriophages and archaeoviruses isolated from environments with a wide range of ionic strengths have been found to be more resistant to variations in ionic strength than their hosts (22). As the cosmopolitan viral families in the Amazon infect bacteria or archaea and as two of them are members of *Caudovirales* (*Myoviridae* and *Podoviridae*) (51), it is plausible that these tailed viruses can move between river and plume. Recent viral metagenomic studies of a rural river in Australia (52) and of the estuary of the Jiulong River in China (53) indicate that *Caudovirales* (e.g., *Myoviridae*, *Siphoviridae*, and *Podoviridae*) were the most abundant viruses. In the Amazon River, members of *Caudovirales* were also abundant, but the higher abundance of *Microviridae*, and also of *Circoviridae*, suggests that the ssDNA families, which are significant pathogens of the phytoplankton and microzooplankton in marine food webs (54), are also very important along the continuum.

The higher viral diversity of the samples from Óbidos, south Macapá, and north Macapá, which have higher Shannon values and rarefaction curves, revealed the effect of different water origins and forest influences, as the main course of the Amazon River receives higher inputs from the forest, upstream waters, and many river tributaries. The enormous export of terrestrial plant and animal material from the Amazon forest into the river may allow certain viral families to proliferate. This phenomenon can be observed on the basis of the abundance of animal- and plant-associated viral families such as the *Circoviridae* that infect animals and of plant viruses such as *Nanoviridae* and *Geminiviridae*, and the data are strengthened by the occurrence of an ssDNA viral genome (Seq_3963), probably related to these viral families, that was abundant and characteristic of riverine waters. Similarly, the virioplanktons of Arctic lakes were dominated by ssDNA viruses such as *Circoviridae* (55), thus reinforcing the idea of the importance of the ssDNA viruses in aquatic environments.

### Dynamics of viral particles and organic matter in the continuum.

Viral structural genes (encoding virion proteins and nucleic acids) and life cycle genes (associated with packaging machinery, phage replication, and phage lysins) were the most abundant in the continuum. However, atypical viral genes (associated with, e.g., cofactors, vitamins, prosthetic groups, and pigments; regulation and cell signaling; cell wall and capsule; fatty acid and lipid biosynthesis; and photosynthesis) were more common in plume and transition localities and also in the Tapajós River, according to the MG-RAST functional profile. The genes that encode other functions in addition to viral structural and nucleic acid replication may be carried by the virions as an effect of the viral horizontal gene transfer. The higher viral particle counts seen in plume locations may enhance the rate of encounters with possible hosts, thus increasing the possibility of transduction processes and subsequently promoting viral diversification. Indeed, the plume, St10 (transition), and Tapajós had more possible viral hosts, according to the reference genomes, which may have led to its more diverse functional profile.

The more-diverse functional profile in the plume was also observed with the most abundant and important viral scaffolds and genomes. The genomes of the viruses from the plume were larger and contained more genes; thus, they can carry more viral enzymatic genes than the compact riverine viral genomes, which might pertain to small ssDNA viruses that have more balanced numbers of viral structural and enzymatic genes. A similar pattern of higher occurrence of large viruses (with respect to capsid and genome size) in estuarine and coastal waters than in freshwater was observed, although small viral particles were dominant along this salinity gradient (56). This trend also explains the identified cellular genes (the majority from *Bacteria*) in these sequences, which were related to basic cellular functions and could represent products of viral transduction in the plume. In addition, one genome carried a protein related to nitrogen fixation (encoded by the *rnf* gene), and three others had oxidoreductases [Fe(II)-dependent oxygenase, tryptophan halogenase, and thioredoxin, which are enzymes that promote oxidative reactions of proteins, forming cascades of signalization], which could represent possible AMGs that help the plume’s viruses during infection, especially in the presence of nitrogen fixation phytoplankton in the plume (14).

Some viral groups may affect the carbon balance in the continuum by infecting photoautotrophic organisms. In rivers, the presence of *Geminiviridae* can facilitate the release of plant organic matter (e.g., lignin and cellulose), which may be degraded by lignocellulolytic bacteria and eukaryotes, being possible drivers of the lignin degradation observed along the river (5, 12, 57). In the plume and in Tapajós, the presence of *Phycodnaviridae* and *Mimiviridae* could decrease the total amount of primary production by their photosynthetic hosts, resulting in less carbon uptake from the atmosphere. The high concentration of humic substances (DOM) in water captures viral particles by adsorption, which reduces viral infectivity in copiotrophic waters, favoring lysogeny (58). The river and the transition plume had DOC values that were 3-fold higher than those seen with the outer plume, likely due to the presence of allochthonous organic matter from the forest. This organic matter and sediment in suspension can adsorb more viral particles, removing them from the water column (30), a process that may be enhanced by the release of extracellular polysaccharides by bacteria and phytoplankton (22). Additionally, the grazing of viral particles is more significant in eutrophic than in oligotrophic waters (30, 59), which may further increase the removal of viruses from copiotrophic river waters. Previous reports showed that freshwater ecosystems tend to have higher VMR (60, 61) or can have similar VMR, as observed in the Charente River, where viral particles counts decreased while salinity increased (62). The possible relation of virioplankton to the presence of organic matter and suspended sediments reported here explains the lower viral counts, especially in the turbid rivers, indicating that the Amazon River has a particular viral-to-microbe ratio dynamics, with a lower VMR in the rivers and a higher VMR in the plume.

Additionally, the pattern showing a lower VMR and lower viral functional Shannon diversity with higher microbial host densities indicates a more lysogenic lifestyle in the river and transition; an opposite scenario was detected in the plume, making the lytic lifestyle more common (19). We thus hypothesize that the widespread changes in water parameters between river and plume may trigger the lytic cycle toward the ocean. Considering this hypothesis, the lack of microbial lysis in the river leaves microbial cells intact for grazing; thus, the organic matter enters the classical food web to nourish higher organisms. In contrast, the lytic lifestyle of the viruses in the plume promotes the viral shunt such that the organic matter is redirected to the microbial communities. However, the suppression of lysis at high microbial cell densities may not be explained by an increase in the prevalence of lysogeny (63). Further studies are needed to elucidate this hypothesis of a lysogenic river and a lytic plume observed here and to perform measurements of the viral contribution to the destiny of organic matter in the Amazon continuum.

### Conclusions.

This is the first study of viromics in the Amazon River continuum to have provided knowledge concerning the diversity and possible ecological roles of viral assemblages in this region. Clear discontinuities were observed throughout the vast Amazon River and plume continuum. Despite the spatial connectivity mediated by the river, the viromes form distinct groups (in rivers, transitions, and plumes), which, together with environmental parameters, indicate that river and plume are different ecosystems. Despite this separation, some bacteriophages are widely distributed throughout the continuum, which indicates that the river-to-ocean transition is a barrier to the distribution of some, but not all, viral families. The viral families are distributed according to a combination of host occurrence and the physical-chemical characteristics of the waters, especially salinity. Knowledge of the current state of the virioplankton of the largest river in the world provides a foundation for understanding how future global warming, or other forms of anthropogenic impact, can influence the microbiota of riverine ecosystems. These changes in microbiota can modify, for example, the river and plume biodiversity and the carbon cycle and sequestration system of the Amazon River continuum, with local (South Atlantic Ocean) and global consequences.

## MATERIALS AND METHODS

### Study location and water sampling.

**FIG 4:**
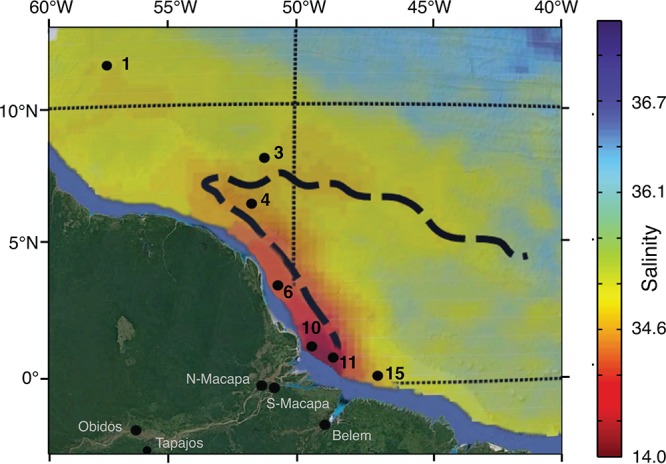
Study area in the Amazon continuum. The study locations included sampling stations in the lower region (continental waters) and in the plume region (Atlantic Ocean) of the Amazon River. The 12 black dots indicate the locations of the sampling stations. River locations, Belém, north Macapá (N-Macapá), south Macapá (S-Macapá), Óbidos, and Tapajós; plume locations (stations), St1, St3, St4, St6, St10, St11, and St15. Main coordinates are also shown. According to Salisbury et al. (95), the traced line indicates the direction of the river plume (mean values, May to July), and the sidebar indicates higher (bluish) and lower (reddish) salinity values.

All sampling was performed in accordance with Brazilian law (ICMBIO no. 33823-1). In both study regions (river and plume) of the Amazon River, water was sampled for metagenomic and flow cytometry analysis. River and plume samples were obtained nearly simultaneously, in a sampling effort that was unprecedented for the Amazon River. The sampling occurred in the period of the year of falling water levels of the Amazon River. River samples were collected from 23 July to 6 August 2012 from the surface waters of each central channel of the following five locations in the lower region of the Amazon River, which comprehends the region between Óbidos and the river mouth (Fig. 4): in Amapá state, north Macapá (0°05.033′S, 51°03.085′W) and south Macapá (0°09.415′S, 50°37.353′W); in Pará state, Belém (1°31.162′S, 48°55.077′W), Óbidos (1°55.141′S, 55°31.543′W), and Tapajós (2°29.063′S, 55°00.450′W). Samples of the Amazon River plume were obtained on board the *RV Atlantis* from 13 to 28 July 2012 from the following seven stations (St) located between the river mouth and the outer plume (Fig. 4): St1 (11°34.241′N, 56°48.354′W), St3 (8°01.335′N, 50°58.92′W), St4 (6°22.320′N, 51°23.625′W), St6 (3°30.331′N, 50°30.002′W), St10 (1°20.285′N, 49°22.263′W), St11 (0°56.959′N, 48°40.425′W), and St15 (0°16.070′N, 47°9.701′W). Surface seawater (0 to 2 m) was collected by gentle impeller pumping (modified Rule 1800 submersible pump) through 10 m of Tygon tubing (inner diameter, 3 cm) and pumped to the ship’s deck, where the water then flowed through a 156-µm-pore-size mesh prefilter and was collected in 20-liter carboys.

### Physical-chemical analyses and chlorophyll measurements.

All environmental parameters were determined using standard riverine or oceanographic methods (5, 64). At least three replicates were analyzed for each parameter. Samples were analyzed for inorganic nutrients (SiO_3_, NO_3_ + NO_2_, and PO_4_) as described previously (65). The values for the fine suspended sediment (FSS) and particulate lignin were obtained from Ward et al. (5).

### Cytometry counts of viral particles and microbial cells.

Triplicate water samples were collected in 2-ml cryogenic vials for each station from the river and the plume. Each of the triplicate samples was fixed with one of the three different preservatives: glutaraldehyde (25% [wt/vol]) for viruses, paraformaldehyde (10% [wt/vol]) for microalgae, or glutaraldehyde plus paraformaldehyde (0.5% [wt/vol] plus 10% [wt/vol]) for bacteria. Cryovials were homogenized, fixed at room temperature for 10 min, and stored in liquid nitrogen. The cytometry counts were performed as described previously (66). The virus-to-microbe ratios (VMR) were also calculated.

### Principal-component analysis (PCA) of the dinucleotide frequency in the metagenomes and the physical-chemical data.

PCA (67) was used to identify the separation patterns between locations prior to the metagenome annotation. Dinucleotide frequencies of the quality-controlled sequences were calculated based on the method described by Willner et al. (68) using homemade Perl scripts (available upon request), as described previously (69). The covariance PCAs of the dinucleotide frequencies and the physical-chemical parameters were performed using R program, version 3.0.2 (70).

### Virome sampling and field processing.

A 40-liter volume of water was sampled in the central channel of each river, while a 100-liter volume was sampled in the plume stations. The total amount of sampled water was prefiltered using a 100-µm-pore-size mesh and was then concentrated to a volume of approximately 0.5 liters using a Tangential Filter Flow (TFF) cassette (GE Healthcare) with a pore size of 100 kDa. The concentrated water was filtered using 3-µm-pore-size mixed cellulose ester membranes (Millipore) to separate larger particulate material and eukaryotic cells and then with 0.22-µm-pore-size polyethersulfone cartridge filters (Sterivex; Millipore) to remove picoplankton cells (bacteria and archaea). The final filtrate (~200 ml) from each site, containing a concentrated fraction of virioplankton, was stored at 4°C in Falcon tubes protected from light until processing in the laboratory was performed, which occurred in a less than a month.

### Virome DNA extraction.

Viral filtrate samples were concentrated by ultracentrifugation, and DNA extraction was performed according to the method described by Gregoracci et al. (71), with the addition of β-mercaptoethanol during lysis and of two washing steps using 10% cetyltrimethylammonium bromide (CTAB) plus 0.7 M NaCl (72). DNA from river samples was additionally cleaned to remove PCR inhibitors, such as residual humic acids, using a OneStep PCR inhibitor removal kit (Zymo Research). Genomiphi reactions were performed using Illustra Genomiphi DNA kit v2 (GE Healthcare) following a modified protocol (73). The DNA concentration was quantified using a NanoDrop ND 1000 spectrophotometer (Thermo Scientific, DE, USA) and a Qubit Fluorometer with a Qubit double-stranded DNA (dsDNA) high-sensitivity (HS) assay kit (Life Technologies, Inc.).

### Illumina library construction and sequencing.

The DNA libraries were prepared using a Nextera XT sample preparation kit (Illumina). The library size distribution was assessed using a model 2100 Bioanalyzer (Agilent) and a High Sensitivity DNA kit (Agilent) and was quantified using an Applied Biosystems 7500 real-time PCR system and a Kapa library quantification kit (Kapa Biosystems). PhiX sequencing control v3 (Illumina) was added at 1%, and paired-end sequencing (2 × 250 bp) was performed on a MiSeq system (Illumina).

### Quality control of the metagenomes and merging of sequences.

Virome reads, in FASTQ files, were submitted to the FastQC project (http://www.bioinformatics.babraham.ac.uk/projects/fastqc) to obtain summary statistics for quality control (QC) of the data sets. The reads were quality filtered (Q Phred, >20) from the results, and artificial duplicated sequences were removed. Twenty bases from the 3′ ends of the reads were then trimmed using the stand-alone version of PRINSEQ (74) to remove low-quality bases. Forward and reverse paired-end sequences with good quality were merged using the SHERA algorithm (75) to extend the size of the obtained reads.

### Virome assembly.

The 12 quality-controlled Amazon viromes were combined, and sequences were cross-assembled using SPAdes (76) with default parameters. Scaffolds larger than 1 kbp were then screened for DNA coding sequences, identification, and initial annotation of proteins, tRNAs, and rRNAs using Prokka (77). Predicted proteins were queried against the NCBI nonredundant protein database with DIAMOND (78) and were annotated taxonomically and functionally according to the best-hit classification (E value, ≤10^−5^).

### Custom viral database and mapping of the Amazon viromes.

The Amazon scaffolds were analyzed with VirSorter (online server, with the metagenome option) (79) to remove scaffolds of possible nonviral origin. Additionally, the assembled scaffolds were also analyzed with VirFinder (45) to compute the likelihood of the assembled sequences being of viral origin through a homology-independent approach. This analysis was performed with the default parameters of VirFinder. A custom viral database was built by adding the VirSorter-validated Amazon viral scaffolds with viral genomes from NCBI viral RefSeq, marine Mediterranean phages obtained from fosmid libraries (80), and prophages mined from bacterial genomes through VirSorter (79). To ensure that this database was nonredundant, the sequences were clustered through BLASTn (81), using values of 95% identity and 40% coverage cutoff. A profile of viral abundance was produced by mapping the raw reads of the 12 Amazon viromes against this custom viral database using Bowtie2 (82) with the -very-sensitive-local and -a options. Ambiguous read counts were corrected as described previously (83). The abundance of each sequence was corrected using the total amount of mapped reads to obtain the relative abundances of viral sequences. Additionally, the complete database (all Amazon scaffolds plus the reference genomes) was also mapped, which generated a complete profile of viral abundance.

A functional analysis of the Amazon viral scaffolds was also performed. First, only the virome reads that matched reference viral genomes or Amazonian scaffolds identified as viral by VirSorter (categories 1 and 2) were selected. Next, these reads were analyzed through the HUMAnN2 (84) analysis pipeline for functional annotation using the uniref90 EC filtered database as the reference. Parameters used to run HUMAnN2 were as follows: humann2—remove-stratified-output—bypass-nucleotide-search—threads 12—evalue 0.001—memory-use maximum—translated-subject-coverage-threshold 0—translated-query-coverage-threshold 20—identity-threshold 30. The HUMAnN2 results with respect to relative abundance levels were categorized according to the corresponding superclasses of MetaCyc (85).

### Statistical analyses and diversity indexes of the Amazon viral scaffolds.

To infer similarities between locations, the mapped profile, based on the custom viral database, was used to build a nonmetric multidimensional scaling (NMDS) ordination, from a Manhattan distance matrix between samples, and a dendrogram, performed with the “hclust” package and “complete linkage” method, both of which were performed in R (70). Based on the mapping only on the reference viral genomes, data corresponding to the host’s groups were obtained and used to infer the occurrence of possible viral hosts.

The abundance profile of the custom viral database was also used in a random forest analysis (86) to determine the most important scaffolds and reference genomes for the separation of river (Tapajós, Óbidos, north Macapá, south Macapá, Belém) and plume (St1, St3, St4, St6, St10, St11, St15) regions. The scaffold and genome architectures were drawn using EasyFig (87), “.gbk” data files from Prokka, and InkScape (http://www.inkscape.org). The possible taxonomical domains of the proteins of the viral genomes were determined by BLASTp (https://blast.ncbi.nlm.nih.gov/Blast.cgi?PAGE=Proteins). All the annotated proteins were classified in terms of their general functions according to the UniProt (http://www.uniprot.org) and Kegg (88) databases.

The mapped profile of the complete database was used to calculate the rarefaction curves, Shannon (89) and Simpson (90) diversity indexes, and richness values, using the R program (70) with the “vegan” package (91).

### Automated taxonomical and functional virome annotation and ordination analysis.

The viromes were submitted to the Metagenomic RAST server (MG-RAST) (92) to obtain summaries of sequence data (metagenome yield, mean sequence size, mean %GC content) and to perform automated taxonomic binning and functional assignment. The 12 metagenomes were classified in MG-RAST against a GenBank database (E value, ≤1e^−5^) which includes Viral RefSeq. Only the sequences that were assigned as pertaining to the viral domain were used for functional analysis against the Subsystems database of MG-RAST, through the “Workbench” tool. The Shannon diversity index of viral functions was calculated using the R program (70) with the “vegan” package (91). The viromes were also analysed using the SILVA database (https://www.arb-silva.de/), through MG-RAST, to assess the number of SSU (small ribosomal subunit) and LSU (large ribosomal subunit) sequences, to evaluate possible cellular contamination.

The ordination of the results of canonical analysis of principal coordinates (CAP) (93) was performed in the R program (70) with the “vegan” package (91). The MG-RAST viral taxonomic matrix was log transformed [log_10_(*x* + 1)], the Bray-Curtis distance was calculated, and the data were compared to a constraint matrix of 10 chosen physical-chemical parameters (Table 1). These constraints were not correlated and were most important for the viral family distribution, based on a “bioenv” analysis, also performed in R. A permutational multivariate analysis of variance (PERMANOVA) (94), based on the Bray-Curtis distance and performed in R, was used to calculate the statistical significance of the CAP ordination data. Considerations regarding the methodological approaches adopted here are reported (see "Caveats" [Text S1 in the supplemental material]).

10.1128/mSphere.00366-17.1 TEXT S1  Caveats. Considerations are presented regarding the methodologies used in the present work, related to issues as viral sampling and viral DNA quality. Download TEXT S1, DOCX file, 0.03 MB. Copyright © 2017 Silva et al. 2017 Silva et al. This content is distributed under the terms of the Creative Commons Attribution 4.0 International license.

### Data availability.

The viromes are available in the MG-RAST server (project “AmazPluma,” number mgp8766) under the following accession numbers: for Belém, mgm4559916.3; for north Macapá, mgm4559917.3; for south Macapá, mgm4559918.3; for Óbidos, mgm4559919.3; for Tapajós, mgm4559927.3; for St1, mgm4559923.3; for St3, mgm4559924.3; for St4, mgm4559925.3; for St6, mgm4559926.3; for St10, mgm4559920.3; for St11, mgm4559921.3; for St15, mgm4559922.3.
