# Supplementary figures and images for: Virioplankton Assemblage Structure in the Lower River and Ocean Continuum of the Amazon
Source: mSphere. 2017 Oct 4;2(5):e00366-17. doi: 10.1128/mSphere.00366-17 (PMC5628290; doi:10.1128/mSphere.00366-17)

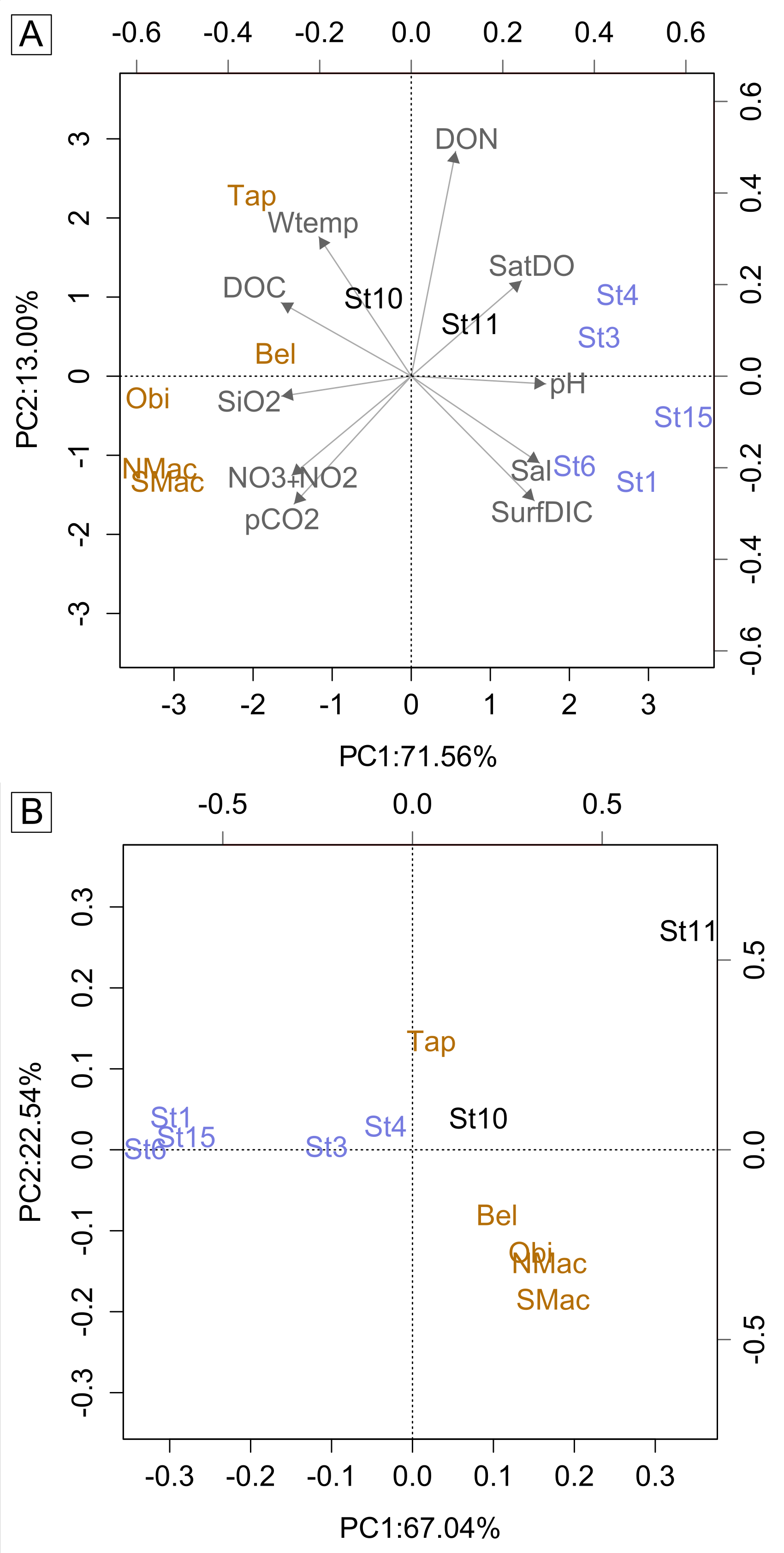

Supplement: FIG S1 [file sph005172371sf1.tif]

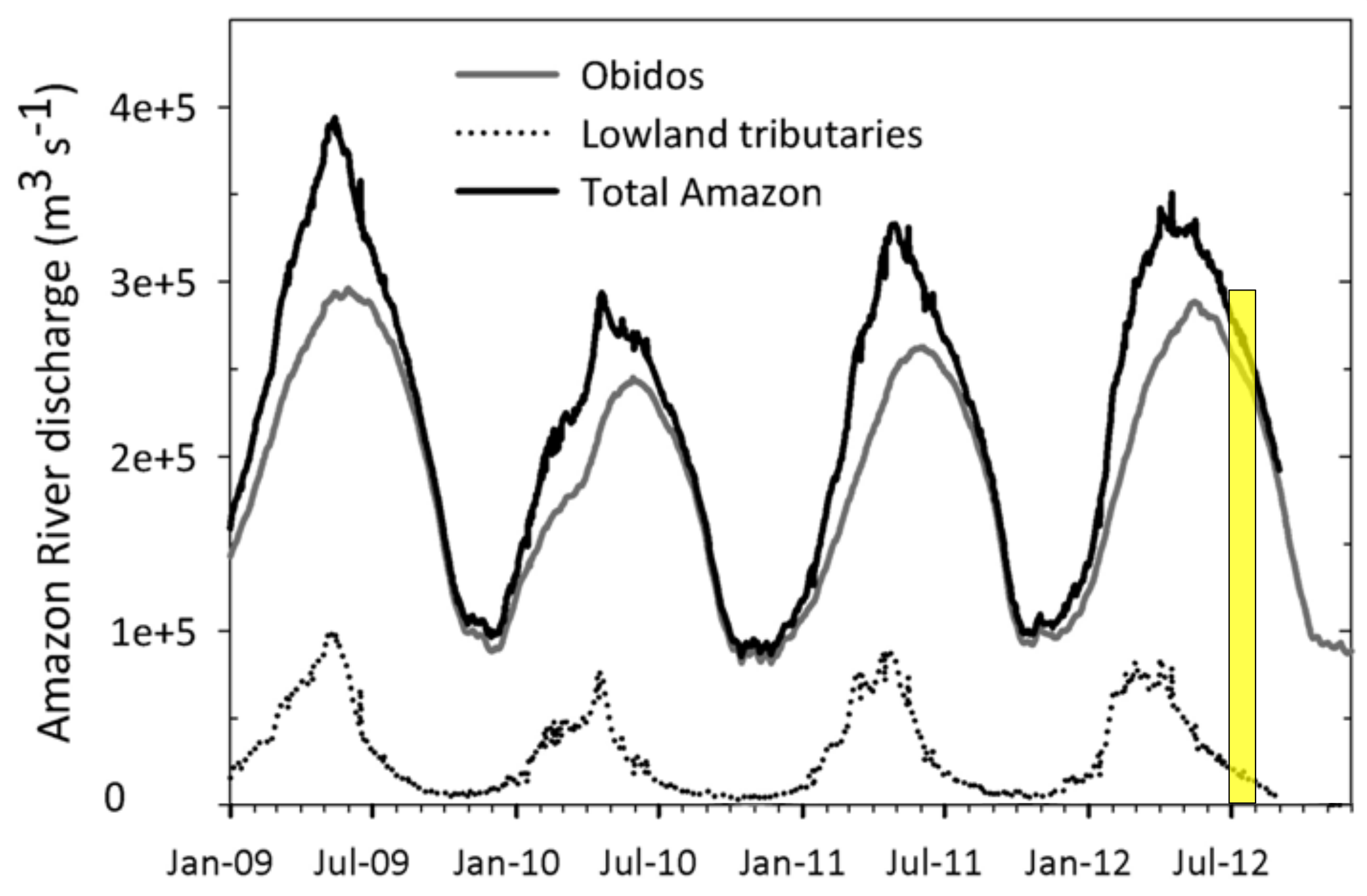

Supplement: FIG S2 [file sph005172371sf2.tif]

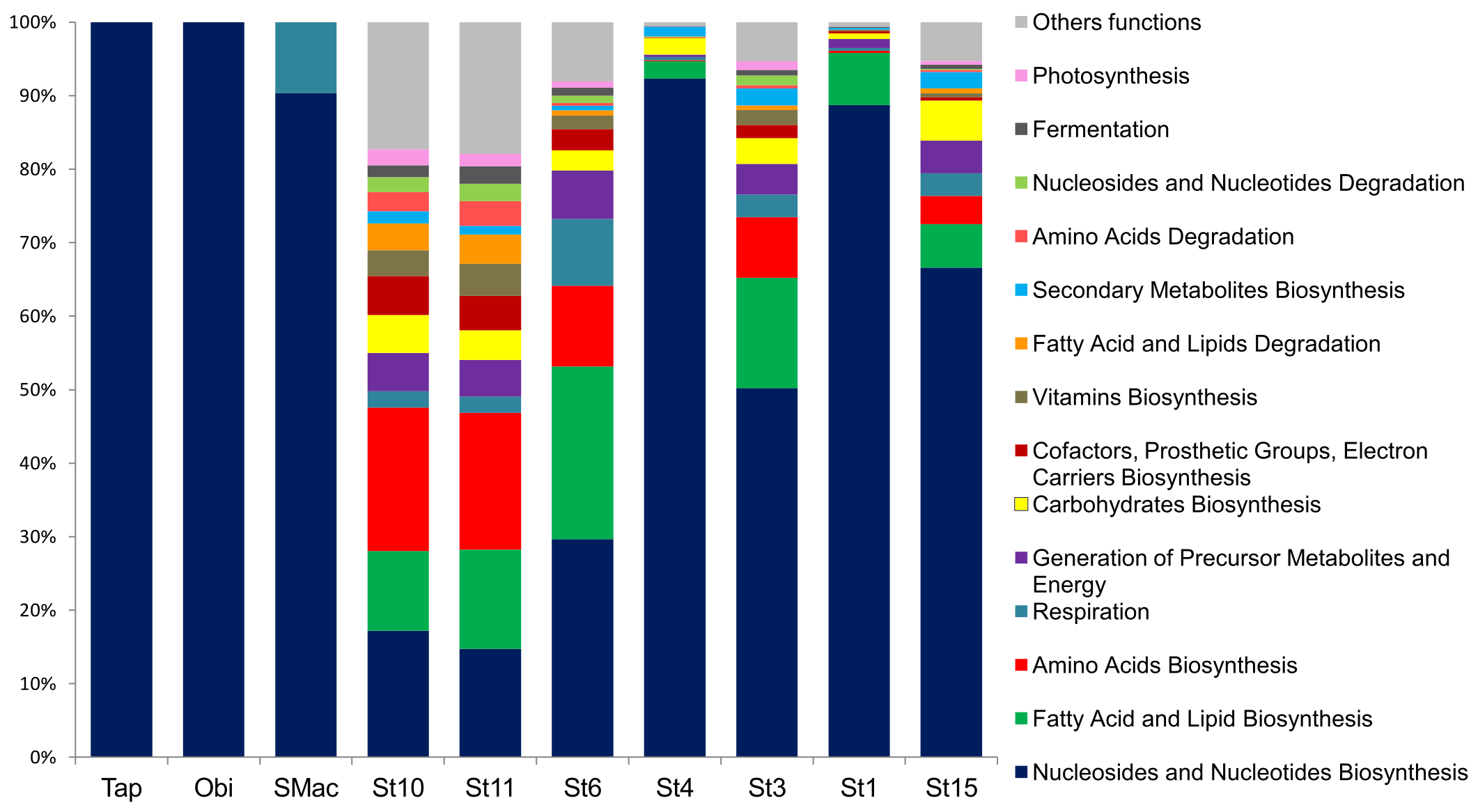

Supplement: FIG S3 [file sph005172371sf3.tif]

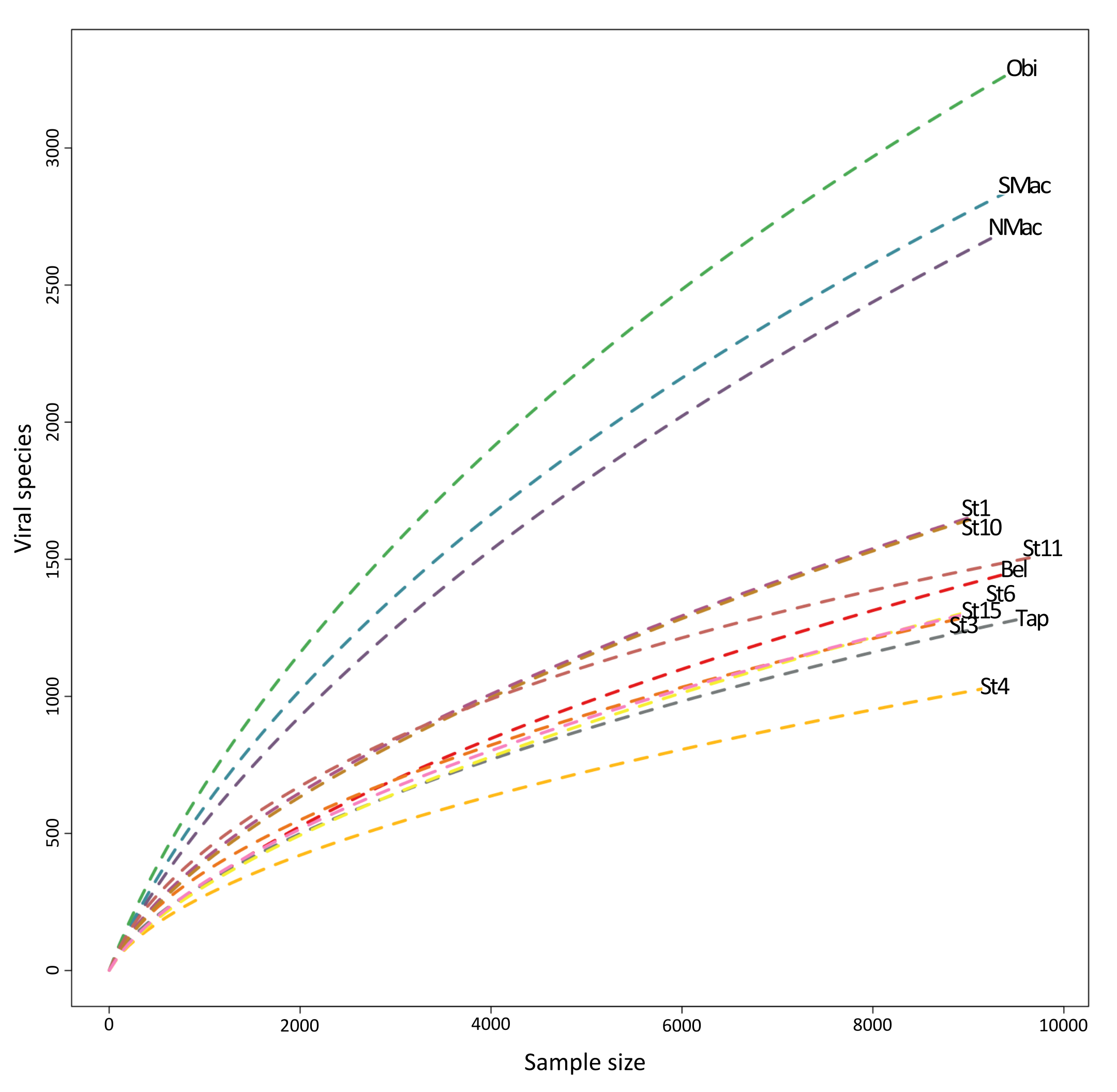

Supplement: FIG S4 [file sph005172371sf4.tif]

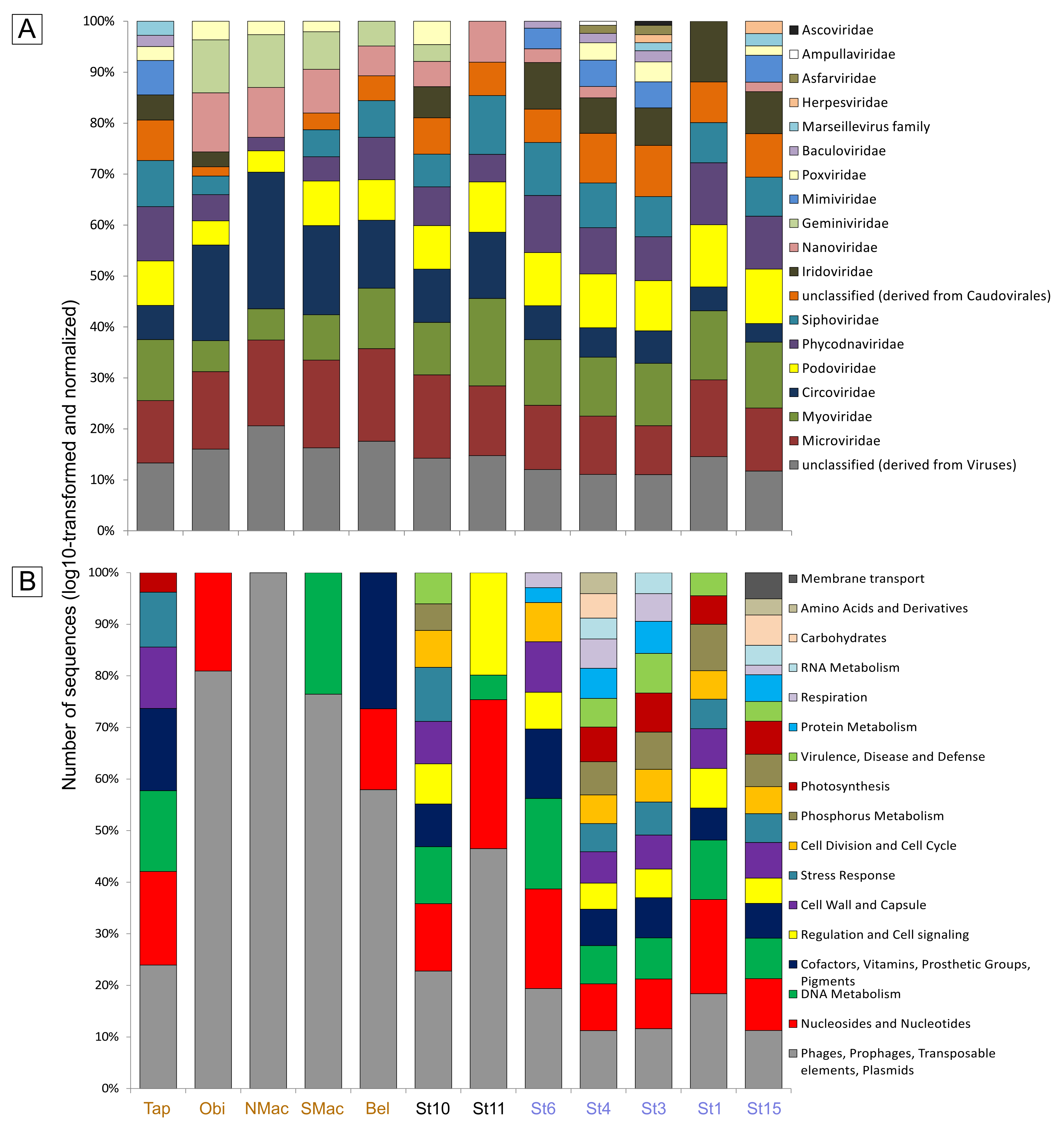

Supplement: FIG S5 [file sph005172371sf5.tif]

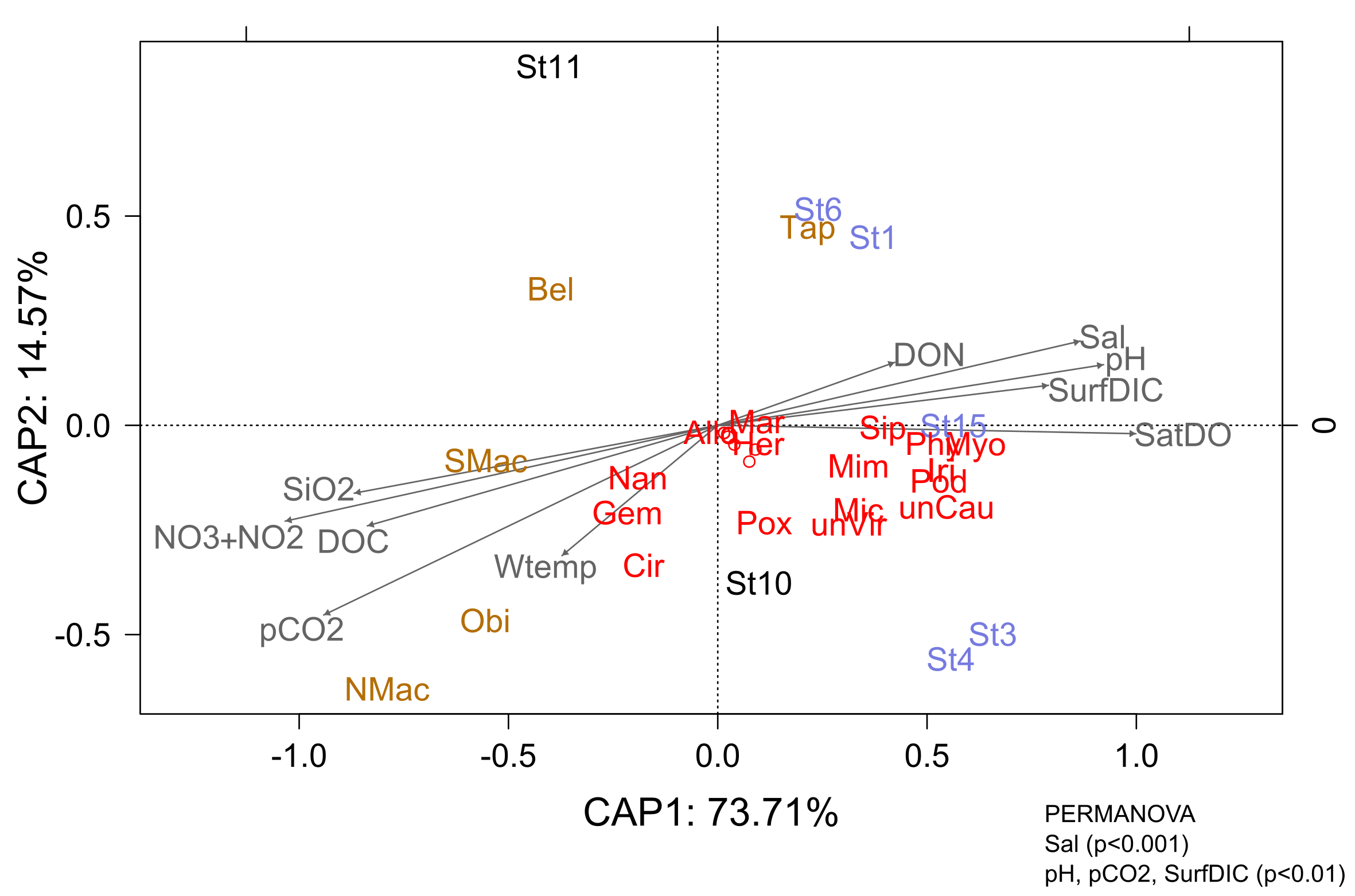

Supplement: FIG S6 [file sph005172371sf6.tif]
