# Supplementary material for: Virioplankton Assemblage Structure in the Lower River and Ocean Continuum of the Amazon
Source: mSphere. 2017 Oct 4;2(5):e00366-17. doi: 10.1128/mSphere.00366-17 (PMC5628290; doi:10.1128/mSphere.00366-17)
Supplement: TABLE S1 [file sph005172371st7.docx]

**Supplemental Table 1**

| **Metagenome**  **(study locations)** | **Tapajós** | **Óbidos** | **North**  **Macapá** | **South**  **Macapá** | **Belém** | **St10** | **St11** | **St6** | **St4** | **St3** | **St1** | **St15** |
| --- | --- | --- | --- | --- | --- | --- | --- | --- | --- | --- | --- | --- |
| **Yield after preprocessing**  **(n^o^ of seqs)** | 342,460 | 306,705 | 502,917 | 215,039 | 295,691 | 867,103 | 146,022 | 628,810 | 2,964,975 | 1,473,246 | 346,240 | 1,531,544 |
| **Mean size of the sequences (bp)** | 211 ± 63 | 295 ± 110 | 208 ± 63 | 359 ± 102 | 210 ± 63 | 211 ± 52 | 238 ± 31 | 223 ± 46 | 218 ± 58 | 195 ± 62 | 172 ± 63 | 222 ± 46 |
| **Mean GC (%)** | 40 ± 10 | 52 ± 9 | 50 ± 10 | 53 ± 9 | 48 ± 12 | 44 ± 10 | 59 ± 7 | 34 ± 6 | 39 ± 8 | 39 ± 9 | 34 ± 7 | 36 ± 7 |
| **Sequences failed**  **to pass the QC**  **n^o^ (% of total seqs)** | 67,407  (19.68) | 48,966  (15.97) | 139,049  (27.65) | 34,767  (16.17) | 107,619  (36.40) | 257,732  (29.72) | 12,608  (8.63) | 166,542  (26.49) | 1,445,857  (48.76) | 447,393  (30.37) | 43,113  (12.45) | 589,809  (38.51) |
| **Sequences passed the QC (valid)**  **n^o^ (% of total seqs)** | 275,053 (80.32) | 257,739 (84.03) | 363,868 (72.35) | 180,272 (83.83) | 188,072  (63.6) | 609,371 (70.28) | 133,414 (91.37) | 462,268 (73.51) | 1,519,118 (51.24) | 1,025,853 (69.63) | 303,127 (87.55) | 941,735 (61.49) |
| **Ribosomal RNA genes**  **n^o^ (% of valid seqs)** | 3,895  (1.42) | 7,144  (2.77) | 9,771  (2.69) | 1,735  (0.96) | 7,632  (4.06) | 17,757  (2.91) | 640  (0.48) | 12,657  (2.74) | 106,346  (7) | 65,502  (6.39) | 17,198  (5.67) | 31,830  (3.38) |
| **Annotated**  **proteins**  **n^o^ (% of valid seqs)** | 40,308  (14.65) | 94,977  (36.85) | 50,544  (13.89) | 46,362  (25.72) | 27,722  (14.74) | 89,936  (14.76) | 96,476  (72.31) | 155,835  (33.71) | 442,203  (29.11) | 417,261  (40.67) | 18,890  (6.23) | 99,160  (10.53) |
| **Unknown**  **proteins**  **n^o^ (% of valid seqs)** | 223,442  (81.24) | 145,103  (56.30) | 287,107  (78.90) | 126,731  (70.30) | 146,419  (77.85) | 488,353  (80.14) | 35,486  (26.6) | 293,776  (63.55) | 970,569  (63.89) | 543,090  (52.94) | 261,538  (86.28) | 810,745  (86.09) |
| **Unknown**  **sequences**  **n^o^ (% of valid seqs)** | 7,408  (2.69) | 10,515  (4.08) | 16,446  (4.52) | 5,444  (3.02) | 6,299  (3.35) | 13,325  (2.19) | 812  (0.61) | 0  (0) | 0  (0) | 0  (0) | 5,501  (1.81) | 0  (0) |
| **Sequences of SSU**  **n^o^ (% of valid seqs)** | 6  (0.002) | 1  (0.000) | 5  (0.001) | 0  (0) | 1  (0.001) | 206  (0.034) | 113  (0.085) | 52  (0.011) | 8  (0.001) | 41  (0.004) | 4  (0.001) | 46  (0.005) |
| **Sequences of LSU**  **n^o^ (% of valid seqs)** | 3  (0.001) | 4  (0.002) | 0  (0) | 0  (0) | 0  (0) | 754  (0.124) | 401  (0.301) | 86  (0.019) | 10  (0.001) | 87  (0.008) | 6  (0.002) | 90  (0.010) |
| **Bacteria (%)** | 73.1 | 31.3 | 33.5 | 43.9 | 49.9 | 63.6 | 99.1 | 94.4 | 32.2 | 40.3 | 50.0 | 60.2 |
| **Viruses (%)** | 17.9 | 61.6 | 61.9 | 46.0 | 47.2 | 35.4 | 0.3 | 2.3 | 60.6 | 55.0 | 38.2 | 30.9 |
| **Eukaryota (%)** | 7.7 | 6.4 | 4.4 | 9.3 | 2.2 | 0.7 | 0.4 | 1.2 | 3.4 | 2.5 | 6.1 | 4.4 |
| **Archaea (%)** | 0.6 | 0.1 | 0.0 | 0.3 | 0.3 | 0.1 | 0.0 | 1.0 | 0.5 | 0.3 | 0.8 | 1.2 |
| **other sequences (%)** | 0.8 | 0.6 | 0.2 | 0.6 | 0.4 | 0.2 | 0.1 | 1.2 | 3.2 | 1.9 | 4.9 | 3.3 |
