# Supplementary material for: Virioplankton Assemblage Structure in the Lower River and Ocean Continuum of the Amazon
Source: mSphere. 2017 Oct 4;2(5):e00366-17. doi: 10.1128/mSphere.00366-17 (PMC5628290; doi:10.1128/mSphere.00366-17)
Supplement: TABLE S3 [file sph005172371st9.docx]

**Supplemental Table 3**

| **Location** | **Shannon** | **Simpson** | **Richness** | **Shannon (functional)** |
| --- | --- | --- | --- | --- |
| Tapajós | 5.500 | 0.984 | 4,407 | 2.95 |
| Óbidos | 7.409 | 0.994 | 7,281 | 1.43 |
| North Macapá | 6.673 | 0.986 | 7,348 | 1.06 |
| South Macapá | 6.793 | 0.976 | 6,822 | 1.50 |
| Belém | 5.298 | 0.969 | 5,749 | 1.45 |
| St10 | 6.095 | 0.981 | 12,707 | 1.42 |
| St11 | 6.151 | 0.992 | 2,751 | 2.28 |
| St6 | 5.047 | 0.920 | 8,094 | 2.68 |
| St4 | 5.162 | 0.951 | 16,846 | 3.08 |
| St3 | 6.072 | 0.981 | 14,951 | 3.09 |
| St1 | 6.116 | 0.985 | 7,533 | 2.34 |
| St15 | 5.263 | 0.942 | 9,743 | 2.92 |
