# Supplementary material for: Virioplankton Assemblage Structure in the Lower River and Ocean Continuum of the Amazon
Source: mSphere. 2017 Oct 4;2(5):e00366-17. doi: 10.1128/mSphere.00366-17 (PMC5628290; doi:10.1128/mSphere.00366-17)
Supplement: TEXT S1 [file sph005172371s10.docx]

**Caveats**

Tangential Flow Filtration is the most widely used method to assess viral diversity (1, 2). In this present study, a large-scale TFF was performed in one single step, and the cellular and viral fractions were separated by manual impact filtration, avoiding further losses caused by a second TFF (3). Viral concentrates were additionally pelleted by ultracentrifugation, and the annotated results were comparable to or even better than other viromes (4). Nevertheless, since the recovery capacity of this protocol has not been quantified, these results may be considered qualitative and not quantitative. Regardless, our protocol could recover the small ssDNA viruses, which were found to be abundant and important in the river continuum. The FeCl_3_ precipitation method to collect viruses (3) has recently been modified for use in a freshwater system (5), but it has not been tested in water bodies filled with suspended particles and organic matter such as the Amazon. Although RNA viruses may be abundant in marine environments (6, 7), the approach used herein focused on DNA viruses. Additionally, although viral samples were subjected to multiple displacement amplification (MDA), its effects are arguable (8, 9); since all samples were subjected to the same protocol, the results remain comparable (5, 10).

Virus collection methodologies usually use a DNAse step to reduce contamination by free non-viral DNA in the sample (5, 10–12). However, the efficiency of this method is not complete, since many ribosomal sequences still remain in the viromes (11, 12). We used the methodology published in Gregoracci and collaborators (4), which did not use DNAse treatment. In addition, we searched for bacterial ribosomal RNA sequences, and the proportions of total rRNA were low, with a maximum of 7% (St4), and at least 0.48% (St11) (Table S1). The search for SSU and LSU recovered even lower data, being less than 0.1% of SSU of the valid sequences, or even zero (SMac); and smaller than 0.3% of LSU, with some samples with zero occurrences (NMac, SMac, Bel) (Table S1). These values are below or even comparable to the levels of non-viral contamination reported in other studies (4, 11, 13). Additionally, the quantitative analysis (NMDS, Dendrogram, possible viral hosts, Random Forest) were all performed with a curated dataset which contains only Amazon viral scaffolds validated with VirSorter (14) and VirFinder tools (15), together with reference viral genomes. Based on these, the profiles obtained were very similar to those obtained with the MG-RAST pipeline, which used all reads, reinforcing that the results presented here are not influenced by cellular contamination.

**References**

1. Thurber R V, Haynes M, Breitbart M, Wegley L, Rohwer F. 2009. Laboratory procedures to generate viral metagenomes. Nat Protoc 4:470–483.

2. Rodriguez-Brito B, Li L, Wegley L, Furlan M, Angly F, Breitbart M, Buchanan J, Desnues C, Dinsdale E, Edwards R, Felts B, Haynes M, Liu H, Lipson D, Mahaffy J, Martin-Cuadrado AB, Mira A, Nulton J, Pasic L, Rayhawk S, Rodriguez-Mueller J, Rodriguez-Valera F, Salamon P, Srinagesh S, Thingstad TF, Tran T, Thurber R V, Willner D, Youle M, Rohwer F. 2010. Viral and microbial community dynamics in four aquatic environments. Isme J 4:739–751.

3. John SG, Mendez CB, Deng L, Poulos B, Kauffman AKM, Kern S, Brum J, Polz MF, Boyle EA, Sullivan MB. 2011. A simple and efficient method for concentration of ocean viruses by chemical flocculation. Environ Microbiol Rep 3:195–202.

4. Gregoracci GB, Dos Santos Soares AC, Miranda MD, Coutinho R, Thompson FL. 2015. Insights into the microbial and viral dynamics of a coastal downwelling-upwelling transition. PLoS One 10:1–14.

5. Dann LM, Rosales S, Mckerral J, Paterson JS, Smith RJ, Jeffries TC, Oliver RL, Mitchell JG. 2016. Marine and giant viruses as indicators of a marine microbial community in a riverine system. Microbiologyopen 1–14.

6. Lang AS, Rise ML, Culley AI, Steward GF. 2009. RNA viruses in the sea. FEMS Microbiol Rev 33:295–323.

7. Steward GF, Culley AI, Mueller J a, Wood-Charlson EM, Belcaid M, Poisson G. 2013. Are we missing half of the viruses in the ocean? ISME J 7:672–9.

8. Yilmaz S, Allgaier M, Hugenholtz P. 2010. Multiple displacement amplification compromises quantitative analysis of metagenomes. Nat Publ Gr 7:943–944.

9. Solonenko S a, Ignacio-Espinoza JC, Alberti A, Cruaud C, Hallam S, Konstantinidis K, Tyson G, Wincker P, Sullivan MB. 2013. Sequencing platform and library preparation choices impact viral metagenomes. BMC Genomics 14:320.

10. Aguirre de Cárcer D, López-Bueno A, Pearce DA, Alcamí A. 2015. Biodiversity and distribution of polar freshwater DNA viruses. Sci Adv 1:e1400127.

11. Hurwitz BL, Brum JR, Sullivan MB. 2015. Depth-stratified functional and taxonomic niche specialization in the “core” and “flexible” Pacific Ocean Virome. ISME J 9:472–484.

12. Ballaud F, Dufresne A, Francez AJ, Colombet J, Sime-Ngando T, Quaiser A. 2016. Dynamics of viral abundance and diversity in a sphagnum-dominated peatland: Temporal fluctuations prevail over habitat. Front Microbiol 6:1–14.

13. Roux S, Krupovic M, Debroas D, Forterre P, Enault F. 2013. Assessment of viral community functional potential from viral metagenomes may be hampered by contamination with cellular sequences. Open Biol 3:130160.

14. Roux S, Enault F, Hurwitz BL, Sullivan MB. 2015. VirSorter: mining viral signal from microbial genomic data. PeerJ 3:e985.

15. Ren J, Ahlgren NA, Lu YY, Fuhrman JA, Sun F. 2017. VirFinder: a novel k-mer based tool for identifying viral sequences from assembled metagenomic data. Microbiome 5:69.
